# Forgoing Healthcare and Insurance Premiums Trends: A 15-Year Population-Based Study in Geneva, Switzerland

**DOI:** 10.3389/ijph.2025.1609027

**Published:** 2025-11-26

**Authors:** Mayssam Nehme, Roxane Dumont, Harris Heritier, Julien Lamour, Shannon Mechoullam, David De Ridder, Idris Guessous

**Affiliations:** 1 Division of Primary Care Medicine, Hôpitaux Universitaires de Genève (HUG), Genève, Switzerland; 2 Faculty of Medicine, University of Geneva, Geneva, Switzerland

**Keywords:** epidemiology, population-based cohort, forgoing care, insurance coverage, socioeconomic determinants

## Abstract

**Objectives:**

Despite compulsory universal health coverage, rising health insurance premiums in Geneva, Switzerland may present financial barriers to care. This study evaluates trends in forgoing healthcare for financial reasons from 2011 to 2025, with associations with insurance premiums, socioeconomic and health-related factors.

**Methods:**

We conducted an annual cross-sectional study with 1,000 randomly selected participants every year (2011–2025), (n = 10,169). The primary outcome was self-reported forgoing of healthcare for financial reasons. Temporal trends and associations with premiums were analyzed using Locally Estimated Scatterplot Smoothing LOESS regression. Logistic regression assessed associations with individual-level determinants.

**Results:**

Overall, 18.1% reported forgoing care, increasing from 15.8% in 2011 to 25.8% in 2025 (+64.6%), corresponding to a 61.9% rise in premiums. Although low income remained a strong predictor (aOR 2.33 [1.76–3.09]), increases were also seen among higher-income groups. Strong correlations were found between premiums and forgoing care, especially in women (0.813) and young adults (0.805).

**Conclusion:**

Additional reforms are needed to reduce growing inequities in access to care despite compulsory universal coverage.

## Introduction

Access to healthcare refers to the timely use of services to achieve the best possible health outcomes [1]. It is an important topic that has long attracted the attention of epidemiologists, physicians, patients and politicians [2–4]. Indeed, timely access to care has been shown to be crucial in preventing disease and disease complication [5, 6]. Additionally, medical needs of patients should be met by allowing them to access care without having to consider forgoing healthcare, especially for financial reasons. While there could be a discourse in choosing whether one should undergo elective procedures or not, access to healthcare should not depend on socioeconomic factors, tending towards a more equitable approach.

Several models have been studied to allow for optimal health coverage. Health coverage takes into account access to care, payments and reimbursements. Premiums and deductibles are considered as payments and are usually tied. When deductibles become too high, an opportunity cost will eventually be calculated by the consumer or patient, and individuals might choose to forgo healthcare to avoid paying the high deductible [7]. Additionally, countries that do not impose mandatory health insurance will tend to attract higher risk individuals to be covered, with potentially average higher costs [8]. On the other hand, individuals with insurance might tend to consume more resources and thus negatively impact costs of healthcare in general [9].

Universal health coverage (UHC) has been presented as one of the solutions to increase insurance coverage and access to care [10]. UHC refers to a system in which all individuals who are eligible have access to a defined set of essential health services without facing financial hardship. However, even in a model like UHC, access to healthcare can still be inequitable and patients might choose to forgo healthcare for financial reasons. In a study evaluating forgoing healthcare in the OECD countries, socioeconomic disparities were evident via especially an income gradient and a difference of over 6 percentage points of forgoing healthcare between the lowest and highest income quintiles [11]. In the United States, 12.1% of adults between 18 and 64 delayed or did not receive needed medical care due to financial reasons [12]. In this same report, forgoing healthcare for financial reasons was associated with socioeconomic disparities.

Switzerland has compulsory health insurance for all its citizens. The model used in Switzerland is consumer-driven with universal health coverage [13]. Health insurance covers the cost of medical treatment including prevention, medication, emergency care services and hospitalizations. The Swiss system combines individual choice with strong public regulation as residents are required to purchase a basic insurance plan from private insurers within a strictly regulated market. Federal law defines a uniform basic benefits package that all insurers must provide. The Federal Office of Public Health (FOPH) supervises insurers, approves annual premium levels. However, as of this date, no public insurance option exists in Switzerland for healthcare coverage.

In Switzerland, a population-based study in 2012 showed that one out of 7 individuals reported forgoing healthcare for economic reasons [14]. Forgoing healthcare was associated with socioeconomic disparities including lower income, female sex, higher deductibles and being a single parent. More recent results on the same study population showed that high deductibles were associated with forgoing healthcare [7] for financial reasons and that dental care was one of the first services to be forgone by patients [15], especially that dental insurance and direct dental care are not subsidized. A recent federal report showed trends in forgoing healthcare in Switzerland [16], however this report only included data up until 2019 (pre-COVID19 pandemic).

The present population-based study relies on data from the canton of Geneva. Geneva represents a highly urbanized region with a diverse population and consistently ranks among the highest cantons in healthcare expenditure *per capita* and mandatory health insurance premiums. To date, no study has evaluated the long-term trends in forgoing healthcare for financial reasons in Switzerland. Given the rise in the costs of healthcare and insurance premiums in Switzerland, and potentially changing behaviors due to the COVID-19 pandemic, it would be essential to assess whether forgoing healthcare for financial reasons has increased with time.

The objective of this study is to look at the trends in forgoing healthcare for financial reasons in the population-based Bus Sante study in Geneva, Switzerland between 2011 and 2025. The study also evaluates associations of forgoing healthcare with socioeconomic or health determinants, as well as the association between forgoing healthcare for financial reasons and health outcomes.

## Methods

The Bus Sante study is a yearly transversal study of about 1’000 participants each year distributed by age and sex representative of the distribution in the general population in Geneva. Participants are randomly selected through lists from the state registry cantonal office of population and migration. Subjects are first contacted via a mailed invitation letter, followed by up to seven phone calls and two more letters. Those who are not reached after these attempts are replaced using the same procedure. Initially, the age range for participants was 35–74 years, but this was expanded to 20–74 years in 2011. The Bus Sante study was briefly interrupted between 2020 and 2023 due to the COVID-19 pandemic and a digitalized version of the questionnaires was introduced in 2024. All participants are French speaking residents of the Geneva canton and have given written informed consent. The study was approved by the Geneva Ethics Committee for Research [IRB00003116].

Overall, n = 10,169 participants were included in this study with an average participation rate of 61%. Participants completed five questionnaires that assessed sociodemographic characteristics, nutrition, physical activity and health outcomes. Participants also underwent a physical examination measuring height, weight and vital signs. Fasting blood samples for a glycemic and lipid profiles were also collected during the visit.

Forgoing healthcare for financial reasons was measured by asking individuals if they had forgone any type of healthcare in the past 12 months for financial reasons, with options “yes” and “no.”

Socioeconomic measures included sex (male, female), age later grouped into age groups (20–34; 35–49; 50–64; 65 and above), education (primary, secondary or tertiary). Civil status was defined as single, married or living in couple, divorced and living alone, divorced and living in couple, widowed and living alone, widowed and living in couple. Nationality was grouped into Swiss and Non-Swiss. Income was measured based on the average monthly income divided into 6 categories in Swiss Francs CHF ≈1.24 USD (<3,000; 3,000–4,999; 5,000–6,999; 7,000–9,499; 9,500–13000; >13,000), individuals could also choose “don’t know”, or “don’t wish to answer”. Healthcare insurance subsidy was measured by asking participants if they receive any subsidy for healthcare insurance with options “yes,” “no,” “don’t know” and “don’t wish to answer.” Individuals reported their health insurance deductibles with options in CHF (None, 300, 500, 1,000, 1,500, 2,000, 2,500, don’t know, and don’t wish to answer). Individuals also reported whether they had a complementary insurance (yes, no).

Health measures included BMI (kg/m2) that was calculated based on measured height and weight and later categorized (underweight, healthy, overweight, obesity), smoking status (yes, no), and self-rated health (very good, good, average, poor, very poor). Diabetes was defined as fasting blood glucose level ≥ 7 mmol/L, self-reported diabetes diagnosis, or self-reported intake of antidiabetic treatment. Diabetes unawareness was defined as blood glucose level ≥ 7 mmol/L, with no reported diagnosis or treatment. Dyslipidemia was defined as fasting cholesterol level ≥6.2 mmol/L, self-reported dyslipidemia diagnosis, or self-reported intake of dyslipidemia treatment. Dyslipidemia unawareness was defined as fasting cholesterol level ≥6.2 mmol/L with no reported diagnosis or treatment. Hypertension was defined as mean systolic and/or diastolic blood pressure ≥140/90 mmHg, self-reported hypertension diagnosis, or self-reported use of anti-hypertensive treatment. Hypertension unawareness was defined as mean systolic and/or diastolic blood pressure ≥140/90 mmHg, with no reported diagnosis or treatment.

Overall trends in insurance premiums (simple market averages) between 2011 and 2025 were collected from the Federal Office of Public Health data [17, 18].

### Statistical Analysis

Continuous variables were expressed as mean with standard deviation (SD). Categorical variables were expressed as number of subjects and percentage. Analyses were performed using Student’s t-tests or ANOVA for continuous data, and chi-square tests for categorical data. Descriptive analyses included the prevalence of forgoing healthcare for financial reasons between 2011 and 2024. Year-trends were analyzed showing the increased percentage of the prevalence of forgoing healthcare for financial reasons and the percentage change in average insurance premiums between 2011 and 2025. Multiple regression models (linear, polynomial, Locally Estimated Scatterplot Smoothing (LOESS), spline, and Holt exponential smoothing) were fitted to estimate the temporal trends in health insurance premiums and forgoing healthcare (Supplementary Table S4). The optimal method for each variable was automatically selected based on Akaike Information Criterion (AIC) to ensure the best model fit while avoiding overfitting. LOESS regression was selected as the optimal model for both variables (Supplementary Table S4). Model validation analysis was also conducted (Supplementary Figure S2). Associations between increase in health insurance premium and the increase in forgoing healthcare were analyzed by sex and by age groups using a scatter plot. The prevalence of forgoing healthcare for financial reasons was stratified by income categories (lowest to highest) between 2011 and 2025. Prevalence was adjusted for age, sex, income and survey year using margins command. Results were presented as predicted prevalence with 95% CI.

Associations between socioeconomic or health determinants and forgoing healthcare for financial reasons were evaluated using logistic regression models. Four models were developed, each focusing on variables related to socioeconomic status, health insurance, general health, and pre-existing conditions. Model 1 included known socioeconomic determinants including age, sex, education, income level, nationality and civil status. Model 2 further included variables related to the insurance coverage including healthcare insurance deductible, subsidy for healthcare insurance, and having complementary insurance. Model 3 integrated additional health determinants including BMI group, smoking, and self-rated health. Model 4 integrated chronic health conditions including diabetes, dyslipidemia and hypertension. Adjustments were done for age, sex, education, income and survey year. Measurements included the adjusted odds ratios with the 95% confidence intervals. An intersectional approach further looked into associations within subgroups (male, female; age 25–34, 35–44, 45–64, 65 and above). A supplementary analysis was done to evaluate associations between socioeconomic or health determinants and forgoing healthcare for financial reasons stratified by deductible (low = 300 to 500 CHF; middle = 1,000–1500 CHF and high = 2000–2500 CHF).

Statistical analysis was conducted using STATA version 16.0 (StataCorp) and R studio (R version 4.4.0).

## Results

### Descriptive Statistics

Out of the 10,169 participants, 51.7% were female, mean age was 48.5 (SD 13) years. Half of participants had a tertiary education level and 10.8% a primary education level. Around 60% of participants were married or living in a couple, while 13.4% were divorced and living alone (10% men and 16.6% women). Income was below 5000 CHF per month in 20% of cases. Health insurance deductibles ranged from 300 CHF (33.8%) to 2500 CHF (22.8%). More women seemed to opt for the lower deductible 38.5% of women had a deductible of 300 CHF *versus* 28.6% of men, and 29.2% of men had a deductible of 2500 CHF compared to 16.9% of women. Overall, 10.8% had a healthcare insurance subsidy. Table 1 shows the baseline characteristics for all participants, and Supplementary Table S1 shows the characteristics by year.

**TABLE 1:** Baseline characteristics of participants (n = 10,169) (Bus Sante study, Geneva, Switzerland, 2011–2025).

Variable	Overall	Male	Female	p-value
	N = 10,169	N = 4,891	N = 5,278	
	N (%)	N (%)	N (%)	
Age, mean (SD)	48.52 (12.99)	48.73 (13.01)	48.32 (12.97)	0.130
Age group				0.400
20–34	1,739 (17.1)	819 (16.7)	920 (17.4)	
35–49	3,916 (38.5)	1,875 (38.3)	2,041 (38.7)	
50–64	3,070 (30.2)	1,476 (30.2)	1,594 (30.2)	
65 and above	1,444 (14.2)	721 (14.7)	723 (13.7)	
Education				0.003
Primary	1,092 (10.8)	504 (10.4)	588 (11.2)	
Secondary	3,658 (36.3)	1,695 (35.1)	1,963 (37.5)	
Tertiary	5,302 (52.6)	2,630 (54.4)	2,672 (51.0)	
Other	20 (0.2)	6 (0.1)	14 (0.3)	
Unknown	97	56	41	
Income level				<0.001
<3,000	656 (6.5)	306 (6.3)	350 (6.6)	
3,000–4,999	1,343 (13.2)	554 (11.3)	789 (14.9)	
5,000–6,999	1,691 (16.6)	755 (15.4)	936 (17.7)	
7,000–9,499	1,871 (18.4)	868 (17.7)	1,003 (19.0)	
9,500–13000	1,800 (17.7)	942 (19.3)	858 (16.3)	
>13,000	2,045 (20.1)	1,178 (24.1)	867 (16.4)	
Don’t know	642 (6.3)	235 (4.8)	407 (7.7)	
Don’t wish to answer	121 (1.2)	53 (1.1)	68 (1.3)	
Civil status				<0.001
Single	1,616 (15.9)	755 (15.5)	861 (16.3)	
Married or living in couple	6,464 (63.7)	3,300 (67.7)	3,164 (60.1)	
Divorced living alone	1,362 (13.4)	486 (10.0)	876 (16.6)	
Divorced living in couple	412 (4.1)	229 (4.7)	183 (3.5)	
Widowed living alone	256 (2.5)	90 (1.8)	166 (3.2)	
Widowed living in couple	33 (0.3)	16 (0.3)	17 (0.3)	
Unknown	26	15	11	
Nationality				0.037
Non-swiss	3,579 (35.3)	1,772 (36.3)	1,807 (34.3)	
Swiss	6,562 (64.7)	3,107 (63.7)	3,455 (65.7)	
Unknown	28	12	16	
Health insurance subsidy				<0.001
No	8,444 (86.5)	4,118 (87.7)	4,326 (85.3)	
Yes	1,053 (10.8)	468 (10.0)	585 (11.5)	
Don’t know	269 (2.8)	108 (2.3)	161 (3.2)	
Don’t wish to answer	1 (0.0)	1 (0.0)	0 (0.0)	
Unknown	402	196	206	
Health insurance deductible				<0.001
None	47 (0.5)	16 (0.3)	31 (0.6)	
300	3,428 (33.8)	1,396 (28.6)	2,032 (38.5)	
500	2,270 (22.4)	936 (19.2)	1,334 (25.3)	
1,000	434 (4.3)	234 (4.8)	200 (3.8)	
1,500	910 (9.0)	520 (10.7)	390 (7.4)	
2000	236 (2.3)	136 (2.8)	100 (1.9)	
2,500	2,318 (22.8)	1,427 (29.2)	891 (16.9)	
Don’t know	487 (4.8)	206 (4.2)	281 (5.3)	
Don’t wish to answer	23 (0.2)	10 (0.2)	13 (0.2)	
Unknown	16	10	6	
BMI group				<0.001
Underweight	939 (9.4)	157 (3.3)	782 (15.0)	
Healthy	4,623 (46.1)	1,992 (41.4)	2,631 (50.5)	
Overweight	3,153 (31.5)	1,960 (40.7)	1,193 (22.9)	
Obesity	1,307 (13.0)	703 (14.6)	604 (11.6)	
Unknown	147	79	68	
Smoking status	2,207 (21.9)	1,177 (24.2)	1,030 (19.7)	<0.001
Self-rated health				0.060
Very good	2,591 (26.7)	1,280 (27.5)	1,311 (26.0)	
Good	5,355 (55.2)	2,592 (55.6)	2,763 (54.8)	
Average	1,559 (16.1)	702 (15.1)	857 (17.0)	
Poor	169 (1.7)	77 (1.7)	92 (1.8)	
Very poor	30 (0.3)	12 (0.3)	18 (0.4)	
Unknown	465	228	237	
Dyslipidemia				<0.001
No	7,393 (73.4)	3,382 (69.7)	4,011 (76.8)	
Yes	2,679 (26.6)	1,467 (30.3)	1,212 (23.2)	
Unknown	97	42	55	
Diabetes				0.024
No	9,513 (94.4)	4,554 (93.9)	4,959 (94.9)	
Yes	559 (5.6)	295 (6.1)	264 (5.1)	
Unknown	97	42	55	
Hypertension				<0.001
No	8,126 (80.6)	3,659 (75.6)	4,467 (85.3)	
Yes	1,951 (19.4)	1,181 (24.4)	770 (14.7)	
Unknown	92	51	41	

**FIGURE 1:**
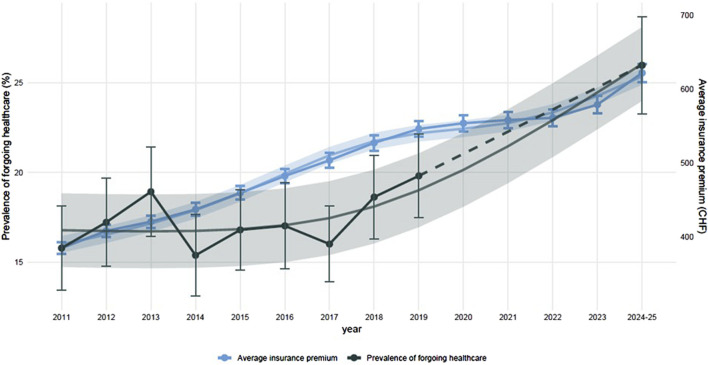
Trends in forgoing healthcare for financial reasons and insurance premiums (Bus Sante study, Geneva, Switzerland, 2011 to 2025). Results show the observed data for the prevalence of forgoing healthcare for financial reasons in the study population, and the yearly average insurance premiums in Geneva between 2011 and 2025 with 95% confidence intervals. Dashed lines correspond to missing data between 2020 and 2023. Trends were estimated using the LOESS model [19].

Overall, 18.1% of participants had forgone healthcare for financial reasons in the 12 months preceding their participation in the study (15.8% in 2011; 25.8% in 2024–25). The prevalence of forgoing healthcare increased by 64.6% with time between 2011 and 2025 (Figure 1), and the trends correlated with a 61.9% increase in health insurance premiums in Switzerland (correlation coefficient: 0.781).

### Trends of Forgoing Healthcare for Financial Reasons

**FIGURE 2:**
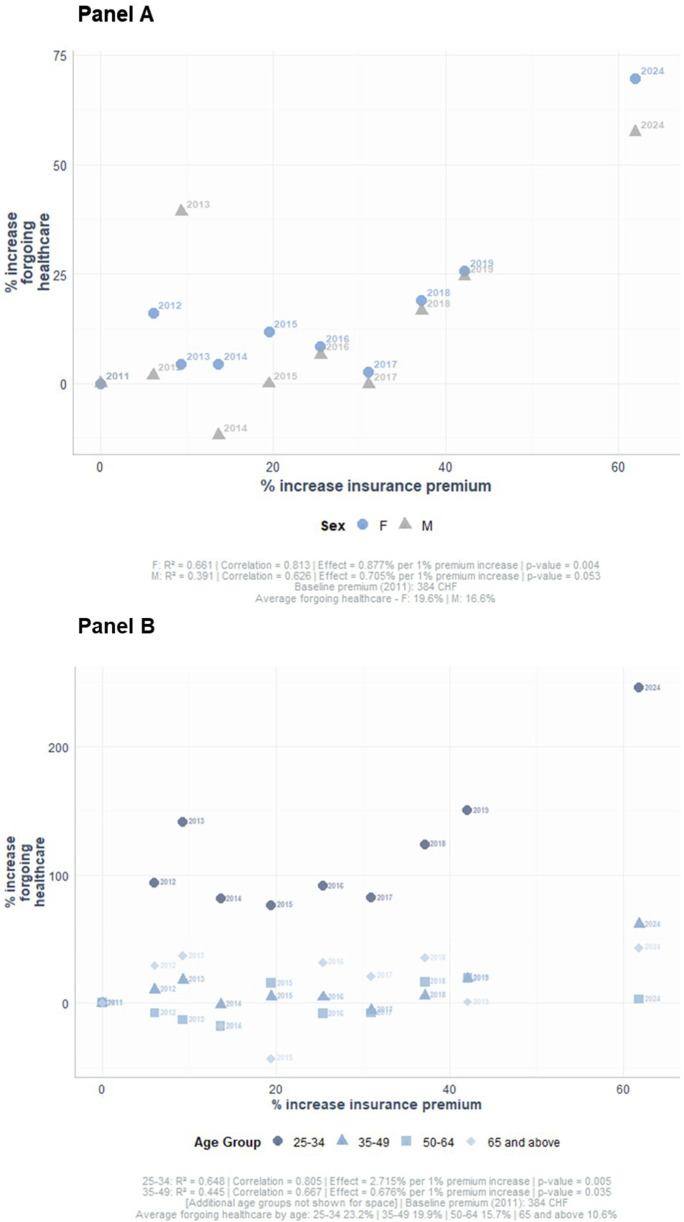
Associations between insurance premium increase and forgoing healthcare by age group and sex (Bus Sante study, Geneva, Switzerland, 2011 to 2025). Results show the correlation between the percent increase in insurance premium and the percent increase in forgoing healthcare for financial reasons between 2011 and 2025 with 95% confidence intervals by sex **(A)** and age groups **(B)**.

For every 10 CHF ≈12.6 USD increase (2% increase) in healthcare premium there was a 2.1% increase in forgoing healthcare. The average prevalence of forgoing healthcare for financial reasons and average changes of premiums between 2011 and 2025 are shown in Supplementary Table S2. The increase in forgoing healthcare was strongly and positively associated with the increase in health insurance premiums (coefficient = 0.781, p-value = 0.004) with 61% of the variance in forgoing healthcare explained by changes in premiums. This association was stronger and more significant in women (coefficient 0.813, p-value = 0.004) compared to men (coefficient 0.626, p-value = 0.053). A stronger correlation was also observed among younger adults (coefficient 0.805, p-value = 0.005) (Figure 2).

Individuals with the lowest incomes had a higher prevalence of forgoing healthcare for financial reasons with a difference ΔPrev of 27.8% between the lowest and highest categories. Trends between 2011 and 2025 additionally show increasingly more individuals forgoing healthcare for financial reasons in the higher income categories (14.0% in 2024–25 compared to 4.5% in 2011). The prevalence of forgoing healthcare for financial reasons by income category is shown in Supplementary Figure S1. After adjusting for age, sex, income and survey year, the predicted prevalence of forgoing healthcare for financial reasons in the group of individuals earning <3000 CHF per month, was 37.0% (24.3–51.3) in 2011 and 27.8% (16.8–40.8) in 2024–25. In the group of individuals earning >13,000 CHF per month, the predicted prevalence of forgoing healthcare for financial reasons was 4.5 (2.1–8.4) in 2011 and 14.0 (6.6–21.5) in 2024–25 (Table 2).

**TABLE 2:** Predicted prevalence of forgoing healthcare by income category (Bus Sante study, Geneva, Switzerland, 2011–2025).

Year	<3,000	3,000–4,999	5,000–6,999	7,000–9,499	9,500–13000	>13,000
2011	37.0 [24.3; 51.3]	23.2 [16.1; 31.6]	21.5 [15.6; 28.4]	14.9 [9.9; 21.0]	10.8 [6.4; 16.8]	4.5 [2.1; 8.4]
2012	35.4 [23.9; 48.2]	34.6 [26.6; 43.2]	16.2 [10.8; 23.0]	16.7 [11.4; 23.2]	9.5 [5.7; 14.6]	2.7 [0.7; 6.8]
2013	29.0 [18.7; 41.2]	33.8 [26.1; 42.2]	20.7 [14.9; 27.5]	15.8 [10.8; 22.0]	15.0 [9.9; 21.3]	5.3 [2.3; 10.2]
2014	34.5 [22.2; 48.6]	26.0 [18.7; 34.3]	20.4 [14.5; 27.3]	9.9 [6.0; 15.3]	13.0 [8.4; 18.9]	6.8 [3.6; 11.5]
2015	48.0 [36.3; 59.8]	20.4 [14.1; 28.0]	21.1 [15.6; 27.6]	12.7 [8.4; 18.2]	10.9 [6.6; 16.7]	6.0 [3.3; 10.1]
2016	27.0 [16.6; 39.7]	26.3 [18.5; 35.4]	19.0 [13.1; 26.1]	18.4 [13.0; 24.9]	12.6 [7.9; 18.8]	8.2 [4.8; 12.8]
2017	35.7 [25.6; 46.9]	22.8 [15.9; 31.1]	20.4 [14.9; 26.8]	17.6 [12.9; 23.3]	14.7 [10.1; 20.5]	4.4 [2.2; 7.7]
2018	40.9 [29.0; 53.7]	31.3 [24.3; 39.0]	26.7 [20.1; 34.1]	14.0 [9.5; 19.6]	11.2 [7.1; 16.4]	7.9 [4.7; 12.4]
2019	33.3 [22.9; 45.2]	24.7 [18.0; 32.4]	23.5 [17.5; 30.4]	22.6 [17.2; 28.9]	17.5 [12.5; 23.6]	10.3 [6.8; 14.7]
2024–25	27.8 [16.8; 40.8]	48.6 [33.5; 56.6]	32.4 [17.5; 47.3]	23.6 [12.0; 35.2]	15.5 [6.6; 24.5]	14.0 [6.6; 21.5]
p-value	<0.001	<0.001	<0.001	<0.001	<0.001	<0.001

Predicted prevalence of forgoing healthcare was adjusted for age, sex, income and survey year.

### Predictors of Forgoing Healthcare for Financial Reasons

When looking at socioeconomic determinants, younger adults were more likely to forgo healthcare for financial reasons compared to individuals 65 years and older. Women were more likely to forgo healthcare for financial reasons (aOR 1.24; 1.09–1.42). Foreign nationality (non-Swiss) was also associated with forgoing healthcare for financial reasons (OR 1.27; 1.15–1.41), however this trend was not significant when adjusted for age, sex, education, income and survey year (aOR 1.04; 0.91–1.19). When compared to being married or in a couple, being divorced and living alone was associated with forgoing healthcare for financial reasons, whereas being single was inversely associated with forgoing healthcare for financial reasons.

Having a lower income was highly associated with forgoing healthcare for financial reasons (aOR 2.33; 1.76–3.09) even after adjusting for age, sex, education level and survey year. Higher deductible categories were associated with an increased risk of forgoing healthcare for financial reasons even after adjusting for the other socioeconomic determinants (aOR 2.06; 1.43–2.98) and (aOR 1.79; 1.51–2.12) for deductible categories of 2000 CHF and 2500 CHF respectively). When stratifying by deductible category (low, middle or high), associations remained mostly similar except for female sex. Women were shown to forgo healthcare for financial reasons when they had a high deductible. Using an intersectional approach, in a subgroup of young adults, forgoing healthcare was not associated with sex, education or nationality, however, was still highly associated with lower income (aOR 2.32; 1.47–3.65). Results were similar when stratifying for each age group. In women, forgoing healthcare for financial reasons was associated with younger age, low income, and being divorced and living alone. When looking at men, the same associations were present for age and income, however there were no associations between civil status and forgoing healthcare in men.

Smoking was associated with forgoing healthcare for financial reasons (aOR 1.43; 1.24–1.65). Having poor or very poor health was also associated with forgoing healthcare for financial reasons with a dose-effect response. Being underweight or overweight was associated with an increase in forgoing healthcare compared to healthy BMI, this association was not found when adjusting for age, sex, education and income. Dyslipidemia and hypertension were associated with lower odds of forgoing healthcare for financial reasons in the unadjusted model, however the association was not significant for dyslipidemia when adjusted for other confounders, whereas it remained significant for hypertension (aOR 0.85; 0.73–0.99). Table 3 shows the associations between socioeconomic determinants, insurance variables, health variables and forgoing healthcare for financial reasons, and Supplementary Table S3 shows these associations stratified by deductible category.

**TABLE 3:** Associations between socioeconomic or health determinants and forgoing healthcare for financial reasons (Bus Sante study, Geneva, Switzerland, 2011–2025).

Variable	Model 1 aOR [95%CI]	p-value	Model 2 aOR [95%CI]	p-value	Model 3 aOR [95%CI]	p-value	Model 4 OR [95%CI]	p-value
Survey year	1.05 [1.04; 1.07]	<0.001	1.07 [1.05; 1.09]	<0.001	1.07 [1.05; 1.09]	<0.001	1.07 [1.05; 1.09]	<0.001
Age groups								
65 years and older	Ref		Ref		Ref		Ref	
25–34	3.51 [2.81–4.39]	<0.001	2.96 [2.31; 3.80]	<0.001	2.95 [2.28; 3.82]	<0.001	2.78 [2.12; 3.65]	<0.001
35; 49	3.04 [2.49; 3.71]	<0.001	2.52 [2.01; 3.15]	<0.001	2.27 [1.80; 2.87]	<0.001	2.15 [1.68; 2.75]	<0.001
50; 64	2.00 [1.63; 2.45]	<0.001	1.78 [1.42; 2.23]	<0.001	1.61 [1.28; 2.03]	<0.001	1.56 [1.23; 1.97]	<0.001
Sex								
Male	Ref		Ref		Ref		Ref	
Female	1.08 [0.97; 1.21]	0.146	1.21 [1.07; 1.37]	<0.001	1.25 [1.10; 1.43]	<0.001	1.24 [1.09; 1.42]	0.001
Income								
7,000; 9,499	Ref		Ref		Ref		Ref	
<3,000	3.15 [2.53; 3.92]	<0.001	2.48 [1.90; 3.24]	<0.001	2.33 [1.76; 3.08]	<0.001	2.33 [1.76; 3.09]	<0.001
3,000; 4,999	2.26 [1.88; 2.72]	<0.001	1.97 [1.61; 2.42]	<0.001	1.88 [1.52; 2.32]	<0.001	1.88 [1.52; 2.33]	<0.001
5,000; 6,999	1.53 [1.28; 1.82]	<0.001	1.34 [1.11; 1.61]	<0.001	1.31 [1.08; 1.59]	0.006	1.31 [1.08; 1.59]	0.006
9,500; 13,000	0.69 [0.57; 0.83]	0.001	0.74 [0.60; 0.90]	0.003	0.74 [0.60; 0.91]	0.004	0.74 [0.60; 0.91]	0.004
>13,000	0.34 [0.27; 0.42]	<0.001	0.38 [0.30; 0.48]	<0.001	0.41 [0.32; 0.52]	<0.001	0.41 [0.32; 0.52]	<0.001
Nationality								
Swiss	Ref		Ref		Ref		Ref	
Non swiss	1.04 [0.92; 1.16]	0.615	1.06 [0.94; 1.21]	0.340	1.04 [0.91; 1.19]	0.579	1.04 [0.91; 1.19]	0.558
Education								
Primary	Ref		Ref		Ref		Ref	
Secondary	1.23 [1.02; 1.48]	0.026	1.28 [1.04; 1.58]	0.020	1.43 [1.15; 1.79]	0.001	1.44 [1.15; 1.80]	0.001
Tertiary	1.35 [1.12; 1.62]	0.001	1.41 [1.14; 1.75]	<0.001	1.68 [1.34; 2.10]	<0.001	1.69 [1.34; 2.11]	<0.001
Civil status								
Married/in couple	Ref		Ref		Ref		Ref	
Single	0.62 [0.53; 0.73]	<0.001	0.64 [0.53; 0.76]	<0.001	0.64 [0.53; 0.77]	<0.001	0.64 [0.53; 0.77]	<0.001
Divorced living alone	1.23 [1.05; 1.44]	0.011	1.26 [1.06; 1.50]	0.009	1.35 [1.13; 1.62]	0.001	1.35 [1.13; 1.62]	0.001
Divorced/in couple	1.09 [0.82; 1.45]	0.544	1.09 [0.80; 1.48]	0.579	1.02 [0.73; 1.42]	0.897	1.03 [0.74; 1.44]	0.858
Widowed living alone	0.84 [0.57; 1.22]	0.356	0.89 [0.59; 1.34]	0.578	0.87 [0.57; 1.32]	0.501	0.86 [0.57; 1.31]	0.487
Widowed/in couple	0.43 [0.10; 1.82]	0.249	0.44 [0.10; 1.89]	0.271	0.44 [0.10; 1.93]	0.274	0.45 [0.10; 1.98]	0.288
Deductible								
300			Ref		Ref		Ref	
500			1.06 [0.90; 1.25]	0.455	1.13 [0.96; 1.34]	0.151	1.13 [0.95; 1.34]	0.159
1,000			1.30 [0.96; 1.75]	0.086	1.33 [0.97; 1.81]	0.074	1.32 [0.97; 1.80]	0.079
1,500			1.29 [1.03; 1.60]	0.024	1.40 [1.11; 1.78]	0.005	1.40 [1.10; 1.77]	0.006
2000			1.67 [1.18; 2.38]	0.004	2.05 [1.42; 2.96]	<0.001	2.06 [1.43; 2.98]	<0.001
2,500			1.52 [1.30; 1.78]	<0.001	1.80 [1.52; 2.12]	<0.001	1.79 [1.51; 2.12]	<0.001
Subsidies								
No			Ref		Ref		Ref	
Yes			1.43 [1.22; 1.67]	0.001	1.42 [1.2; 1.68]	<0.001	1.42 [1.20; 1.68]	<0.001
Complementary insurance								
Yes			Ref		Ref		Ref	
No			1.67 [1.47; 1.90]	<0.001	1.58 [1.39; 1.81]	<0.001	1.59 [1.39; 1.82]	<0.001
Smoking								
No					Ref		Ref	
Yes					1.44 [1.25; 1.65]	<0.001	1.43 [1.24; 1.65]	<0.001
Self-rated health								
Very good					Ref		Ref	
Good					1.54 [1.31; 1.81]	<0.001	1.55 [1.32; 1.82]	<0.001
Average					2.82 [2.31; 3.44]	<0.001	2.84 [2.33; 3.46]	<0.001
Poor					5.18 [3.40; 7.90]	<0.001	5.15 [3.37; 7.85]	<0.001
Very poor					5.8 [2.08; 16.17]	<0.001	5.65 [2.01; 15.86]	<0.001
BMI								
Healthy					Ref		Ref	
Underweight					1.11 [0.90; 1.36]	0.335	1.10 [0.90; 1.36]	0.317
Overweight					1.03 [0.89; 1.20]	0.673	1.04 [0.90; 1.21]	0.662
Obesity					1.12 [0.92; 1.37]	0.245	1.17 [0.95; 1.43]	0.053
Dyslipidemia								
No							Ref	
Yes							0.85 [0.73; 0.99]	0.044
Diabetes								
No							Ref	
Yes							1.27 [0.96; 1.67]	0.084
Hypertension								
No							Ref	
Yes							0.83 [0.69:1.01]	0.062

Model 1: adjusted for demographics (age, sex, income, nationality, education) and survey year.

Model 2: model 1 + insurance variables (healthcare insurance premium, insurance subsidies, complementary insurance).

Model 3: model 2 + health variables (smoking, self-rated health, body mass index BMI).

Model 4: model 3 + chronic conditions (dyslipidemia, diabetes, hypertension).

## Discussion

In 2025, one out of four individuals in a population-based study in Geneva, Switzerland reported forgoing healthcare for financial reasons. Trends between 2011 and 2025 show an increase in forgoing healthcare along increasing insurance premiums, with a 2% increase in forgoing healthcare for financial reasons for every 10 CHF (12.6 USD) increase in insurance premiums. Forgoing healthcare for financial reasons increased in higher income categories, with increasingly more higher income individuals forgoing healthcare for financial reasons (+9.5%). Results revealed a strong and positive correlation between the increase in health insurance premiums and the rise in forgoing healthcare, with nearly 61% of the variance explained by premium increases alone. Results showed that women and young adults were disproportionately burdened by rising healthcare costs. Young adults could perceive themselves as healthier and have more risk tolerance in forgoing healthcare for financial reasons. Women, individuals with a lower income, divorced individuals living alone, as well as smokers, individuals suffering from obesity or underweight, and individuals with poorer self-rated health were more likely to forgo healthcare for financial reasons.

Forgoing healthcare for financial reasons is an important determinant in access to care in a timely manner. When individuals delay or forgo care due to cost, they become at increased risk of finding disease later in its progression, with higher severity, more complications and an increase in avoidable hospitalizations. A pooled study in 2020 showed that 17.9% of households had forgone healthcare and 10.3% in 2021 [20]. However, these results were evaluated during the COVID-19 pandemic and it is important to look beyond the pandemic years, especially with half of the world’s population lacking access to essential health services in 2024 [21], despite the push towards universal healthcare coverage, results shows that even within this model, disparities persist and 25% of individuals forgo healthcare for financial reasons. With an increasing prevalence of forgoing healthcare for financial reasons in Geneva, Switzerland, the population and healthcare system are at risk of incurring increased costs, and deteriorating healthcare. A projected further rise in forgoing healthcare highlights the need to reevaluate the healthcare system based on increasing health insurance premiums.

Results showed that lower income was significantly associated with forgoing healthcare for financial reasons. However, more detailed results also showed that individuals with a higher income were forgoing healthcare for financial reasons differentially more than individuals with a lower income, between 2011 and 2025. This phenomenon could be explained by increasing premiums, but also by an increase in the subsidies for healthcare insurance in lower income groups. Indeed, in Geneva recent years have shown an increase in subsidies for health insurance for lower income groups [22].

The observed increase in forgoing healthcare and correlation with rising insurance premiums shows that affording healthcare insurance is a growing concern across the income spectrum, despite subsidies being allocated to those with lower income. With increasing premiums and financial considerations from a household perspective for several family members, individuals might be more likely to choose high-deductible plans for their household. High deductibles, one of the options to decrease an individual’s insurance premiums, could be a significant factor in forgoing healthcare for financial reasons due to out-of-pocket expenses [7], and a potential target for policymakers. Results in this study showed that the association between an increase in deductibles and forgoing healthcare for financial reasons remained significant even after adjusting for all other socioeconomic factors including age, sex, education and income.

Associations showed that younger adults and women were more likely to forgo healthcare for financial reasons. A positive strong correlation was seen between forgoing healthcare and the increase in health insurance premiums especially in women and young adults with respectively 66% and 65% of the variance explained by premium increases alone. After adjusting for income, health insurance premiums, and subsidies, younger age was still independently associated with higher odds ratios of forgoing healthcare for financial reasons. It is possible that younger adults perceive themselves as generally healthier and allow themselves more margins to forgo healthcare for different reasons including financial reasons. The strong correlation between the increase in forgoing healthcare and the increase in premiums in this population group warrants a more targeted approach and potentially more attractive premiums for this population. Women were also consistently more likely to forgo healthcare for financial reasons after multivariable adjustments for other socioeconomic and health determinants, and that was especially true in the subgroup of women with high deductibles. This could be due to a socioeconomic disparity between men and women, but also to caregiving responsibilities falling largely on women and delaying access to care [23]. Divorced individuals living alone were also more likely to forgo healthcare for financial reasons, the financial burden of being a single parent or being divorced and living alone, without shared household resources could accelerate forgoing healthcare. Interestingly, this association was seen in women and not in men, further highlighting sex-based socioeconomic disparity.

Health variables such as smoking and being in poor health were associated with forgoing healthcare for financial reasons. Smokers are potentially individuals that put less emphasis on their health by engaging in deleterious behaviors and potentially forgoing healthcare. This would create a double burden of high-risk behavior and poorer access to care, leading to undiagnosed chronic conditions and long-term complications. Similarly, individuals with poor or very poor health were more likely to forgo healthcare for financial reasons, suggesting that those who need healthcare the most might not be accessing it. This phenomenon has been described in the literature as inverse care law [24–26], creating a differentially higher burden for individuals with poor health. Some of the protective associations seen with dyslipidemia and hypertension, where individuals who suffered from these conditions seemed to be less likely to forgo healthcare for financial reasons, could reflect higher contact with the healthcare system for individuals with chronic conditions, especially for those who are being managed. While this model could indicate that healthcare access is met when needed (pre-existing conditions), reducing potential overconsumption, it is important to note that individuals with poor self-rated health still had increased levels of forgoing healthcare for financial reasons, potentially worsening their health. Self-rated health has been shown in the literature to be associated with mortality [27].

The study has several limitations including self-reported data that could be subject to recall bias and social desirability bias. However, this is partly mitigated by the large sample size. Second, while the population-based recruitment allows for a sample that is representative of the general population, non-respondents could have different health behaviors including access to care. This study included French speaking residents of the canton of Geneva, thus potentially excluding non-francophone migrant groups. Adjusting for age, sex, education and income partly addressed this limitation. Third, data were missing between 2020 and 2023, and more long-term data over the next few years are needed to evaluate whether trends are fluctuating or more linear. While this is important to keep in mind, the increase between 2011 and 2025 was already evident, and calls for close monitoring.

### Conclusion

Forgoing healthcare for financial reasons has considerably increased between 2011 and 2025, in a population-based study in Geneva, Switzerland, where universal health coverage is compulsory. While traditional socioeconomic factors continue to influence this trend, findings also show a growing prevalence of forgoing healthcare in middle- and high-income groups correlated with a substantial increase in insurance premiums. Healthcare systems should find the right balance between addressing healthcare needs while limiting overconsumption. Addressing these challenges requires broader long-term strategies considering the rate of increase in insurance premiums. Targeting more vulnerable groups including younger adults and women by offering them advantageous health insurance plans could help reduce the potential barriers to care and is necessary to allow for better health and healthcare access for all.

## Supplementary Material

SUPPLEMENTARY TABLE S1 Baseline characteristics of participants by year (n = 10,169) (Bus Sante study, Geneva, Switzerland. 2011 to 2025).

SUPPLEMENTARY TABLE S2 Average prevalence of forgoing healthcare for financial reasons, with average changes of premiums (Bus Sante study, Geneva, Switzerland, 2011 to 2025).

SUPPLEMENTARY TABLE S3 Associations between socioeconomic or health determinants and forgoing healthcare for financial reasons stratified by deductible (Bus Sante study, Geneva, Switzerland, 2011 to 2025).

SUPPLEMENTARY TABLE S4 Model performance comparison for predictions of changes in insurance premium and forgoing healthcare (Bus Sante study, Geneva, Switzerland, 2011 to 2025).

SUPPLEMENTARY FIGURE S1 Trends of forgoing healthcare for financial reasons stratified by income categories (Bus Sante study, Geneva, Switzerland, 2011 to 2025).

SUPPLEMENTARY FIGURE S2 LOESS Model validation analysis (Bus Sante study, Geneva, Switzerland, 2011 to 2025).
