# Supplementary material for: Forgoing Healthcare and Insurance Premiums Trends: A 15-Year Population-Based Study in Geneva, Switzerland
Source: Int J Public Health. 2025 Nov 26;70:1609027. doi: 10.3389/ijph.2025.1609027 (PMC12689442; doi:10.3389/ijph.2025.1609027)
Supplement: Supplementary file 1 [file Supplementaryfile1.docx]

**Supplementary Table 1. Baseline characteristics of participants by year (n=10,169) (Bus Sante study, Geneva, Switzerland. 2011 to 2025)**

| **variable** | **Overall**, N = 10,169*^1^* | **2011**  N = 942*^1^* | **2012**  N = 913*^1^* | **2013**  N = 947*^1^* | **2014**  N = 973*^1^* | **2015**  N = 1,077*^1^* | **2016**  N = 953*^1^* | **2017**  N = 1,157*^1^* | **2018**  N = 1,075*^1^* | **2019**  N = 1,117*^1^* | **2024/25**  N = 1,015*^1^* | **p-value***^2^* |
| --- | --- | --- | --- | --- | --- | --- | --- | --- | --- | --- | --- | --- |
| **Age, mean(SD)** | 48.52 (12.99) | 51.58(10.78) | 49.43(13.14) | 47.82(13.31) | 48.07(12.96) | 48.41(13.01) | 47.97(13.20) | 48.16(12.96) | 49.48(12.97) | 48.44(13.20) | 46.05(13.42) | <0.001 |
| **Age group** |  |  |  |  |  |  |  |  |  |  |  |  |
| 25;34 | 1,739 (17.1%) | 9 (1.0%) | 145 (15.9%) | 194 (20.5%) | 194 (19.9%) | 190 (17.6%) | 181 (19.0%) | 213 (18.4%) | 165 (15.3%) | 198 (17.7%) | 250 (24.6% |  |
| 35;49 | 3,916 (38.5%) | 467 (49.6%) | 356 (39.0%) | 347 (36.6%) | 349 (35.9%) | 425 (39.5%) | 367 (38.5%) | 453 (39.2%) | 383 (35.6%) | 401 (35.9%) | 368 (36.3%) |  |
| 50;64 | 3,070 (30.2%) | 326 (34.6%) | 263 (28.8%) | 273 (28.8%) | 313 (32.2%) | 307 (28.5%) | 265 (27.8%) | 332 (28.7%) | 361 (33.6%) | 348 (31.2%) | 282 (27.8%) |  |
| 65 and above | 1,444 (14.2%) | 140 (14.9%) | 149 (16.3%) | 133 (14.0%) | 117 (12.0%) | 155 (14.4%) | 140 (14.7%) | 159 (13.7%) | 166 (15.4%) | 170 (15.2%) | 115 (11.3%) |  |
| **Sex** |  |  |  |  |  |  |  |  |  |  |  | **0.9** |
| Female | 5,278 (51.9%) | 483 (51.3%) | 450 (49.3%) | 500 (52.8%) | 514 (52.8%) | 548 (50.9%) | 497 (52.2%) | 602 (52.0%) | 558 (51.9%) | 594 (53.2%) | 532 (52.4%) |  |
| Male | 4,891 (48.1%) | 459 (48.7%) | 463 (50.7%) | 447 (47.2%) | 459 (47.2%) | 529 (49.1%) | 456 (47.8%) | 555 (48.0%) | 517 (48.1%) | 523 (46.8%) | 483 (47.6%) |  |
| **Education** |  |  |  |  |  |  |  |  |  |  |  |  |
| Primary | 1,092 (10.8%) | 106 (11.5%) | 115 (12.8%) | 127 (13.6%) | 137 (14.2%) | 132 (12.4%) | 83 (8.8%) | 111 (9.7%) | 101 (9.4%) | 88 (7.9%) | 92 (9.1%) |  |
| Secondary | 3,658 (36.3%) | 406 (44.0%) | 340 (37.9%) | 360 (38.7%) | 356 (36.9%) | 410 (38.4%) | 359 (37.9%) | 396 (34.7%) | 394 (36.7%) | 368 (32.9%) | 269 (26.6%) |  |
| Tertiary | 5,302 (52.6%) | 411 (44.5%) | 443 (49.3%) | 444 (47.7%) | 471 (48.9%) | 526 (49.3%) | 505 (53.3%) | 634 (55.6%) | 578 (53.9%) | 661 (59.2%) | 629 (62.3%) |  |
| Other | 20 (0.2%) | 0 (0.0%) | 0 (0.0%) | 0 (0.0%) | 0 (0.0%) | 0 (0.0%) | 0 (0.0%) | 0 (0.0%) | 0 (0.0%) | 0 (0.0%) | 20 (2.0%) |  |
| Unknown | 97 | 19 | 15 | 16 | 9 | 9 | 6 | 16 | 2 | 0 | 5 |  |
| **Income** |  |  |  |  |  |  |  |  |  |  |  | **<0.001** |
| <3000 | 656 (6.5%) | 54 (5.7%) | 65 (7.1%) | 69 (7.3%) | 55 (5.7%) | 75 (7.0%) | 64 (6.7%) | 84 (7.3%) | 66 (6.1%) | 75 (6.7%) | 49 (4.8%) |  |
| 3000;4999 | 1,343 (13.2%) | 126 (13.4%) | 136 (14.9%) | 142 (15.0%) | 131 (13.5%) | 142 (13.2%) | 115 (12.1%) | 127 (11.0%) | 163 (15.2%) | 150 (13.4%) | 111 (10.9%) |  |
| 5000;6999 | 1,691 (16.6%) | 172 (18.3%) | 155 (17.0%) | 174 (18.4%) | 168 (17.3%) | 197 (18.3%) | 153 (16.1%) | 191 (16.5%) | 165 (15.3%) | 179 (16.0%) | 137 (13.5%) |  |
| 7000;9499 | 1,871 (18.4%) | 175 (18.6%) | 168 (18.4%) | 177 (18.7%) | 181 (18.6%) | 199 (18.5%) | 180 (18.9%) | 221 (19.1%) | 200 (18.6%) | 212 (19.0%) | 158 (15.6%) |  |
| 9500;13000 | 1,800 (17.7%) | 159 (16.9%) | 190 (20.8%) | 167 (17.6%) | 177 (18.2%) | 166 (15.4%) | 160 (16.8%) | 198 (17.1%) | 198 (18.4%) | 194 (17.4%) | 191 (18.8%) |  |
| >13000 | 2,045 (20.1%) | 200 (21.2%) | 147 (16.1%) | 150 (15.8%) | 177 (18.2%) | 218 (20.2%) | 210 (22.0%) | 250 (21.6%) | 214 (19.9%) | 253 (22.6%) | 226 (22.3%) |  |
| Don't know | 642 (6.3%) | 56 (5.9%) | 52 (5.7%) | 68 (7.2%) | 84 (8.6%) | 80 (7.4%) | 71 (7.5%) | 86 (7.4%) | 45 (4.2%) | 33 (3.0%) | 67 (6.6%) |  |
| Don't wish to answer | 121 (1.2%) | 0 (0.0%) | 0 (0.0%) | 0 (0.0%) | 0 (0.0%) | 0 (0.0%) | 0 (0.0%) | 0 (0.0%) | 24 (2.2%) | 21 (1.9%) | 76 (7.5%) |  |
| **Civil status** |  |  |  |  |  |  |  |  |  |  |  |  |
| Single | 1,616 (15.9%) | 80 (8.5%) | 141 (15.5%) | 163 (17.2%) | 166 (17.1%) | 152 (14.1%) | 162 (17.0%) | 188 (16.2%) | 163 (15.2%) | 194 (17.4%) | 207 (20.8%) |  |
| Married/in couple | 6,464 (63.7%) | 609 (64.7%) | 560 (61.4%) | 604 (63.8%) | 604 (62.1%) | 697 (64.8%) | 612 (64.2%) | 742 (64.1%) | 684 (63.6%) | 699 (62.6%) | 653 (65.8%) |  |
| Divorced living alone | 1,362 (13.4%) | 152 (16.2%) | 127 (13.9%) | 116 (12.2%) | 132 (13.6%) | 158 (14.7%) | 118 (12.4%) | 142 (12.3%) | 155 (14.4%) | 146 (13.1%) | 116 (11.7%) |  |
| Divorced/in couple | 412 (4.1%) | 67 (7.1%) | 56 (6.1%) | 44 (4.6%) | 45 (4.6%) | 51 (4.7%) | 39 (4.1%) | 64 (5.5%) | 16 (1.5%) | 30 (2.7%) | 0 (0.0%) |  |
| Widowed living alone | 256 (2.5%) | 27 (2.9%) | 22 (2.4%) | 17 (1.8%) | 24 (2.5%) | 17 (1.6%) | 19 (2.0%) | 15 (1.3%) | 54 (5.0%) | 44 (3.9%) | 17 (1.7%) |  |
| Widowed/in couple | 33 (0.3%) | 6 (0.6%) | 6 (0.7%) | 3 (0.3%) | 1 (0.1%) | 1 (0.1%) | 3 (0.3%) | 6 (0.5%) | 3 (0.3%) | 4 (0.4%) | 0 (0.0%) |  |
| Unknown | 26 | 1 | 1 | 0 | 1 | 1 | 0 | 0 | 0 | 0 | 22 |  |
| **Swiss nationality** |  |  |  |  |  |  |  |  |  |  |  | <0.001 |
| Non;Swiss | 3,579 (35.3%) | 295 (31.4%) | 303 (33.4%) | 322 (34.0%) | 377 (38.8%) | 422 (39.3%) | 350 (36.8%) | 412 (35.6%) | 344 (32.0%) | 388 (34.7%) | 366 (36.6%) |  |
| **variable** | **Overall**, N = 10,169*^1^* | **2011**  N = 942*^1^* | **2012**  N = 913*^1^* | **2013**  N = 947*^1^* | **2014**  N = 973*^1^* | **2015**  N = 1,077*^1^* | **2016**  N = 953*^1^* | **2017**  N = 1,157*^1^* | **2018**  N = 1,075*^1^* | **2019**  N = 1,117*^1^* | **2024/25**  N = 1,015*^1^* | **p-value***^2^* |
| Swiss | 6,562 (64.7%) | 645 (68.6%) | 605 (66.6%) | 624 (66.0%) | 595 (61.2%) | 653 (60.7%) | 601 (63.2%) | 745 (64.4%) | 731 (68.0%) | 729 (65.3%) | 634 (63.4%) |  |
| Unknown | 28 | 2 | 5 | 1 | 1 | 2 | 2 | 0 | 0 | 0 | 15 |  |
| **Health insurance subsidy** |  |  |  |  |  |  |  |  |  |  |  |  |
| No | 8,444 (86.5%) | 768 (85.3%) | 748 (87.0%) | 752 (83.4%) | 797 (85.9%) | 897 (86.1%) | 790 (86.4%) | 956 (85.4%) | 897 (87.3%) | 928 (87.5%) | 911 (89.9%) |  |
| Yes | 1,053 (10.8%) | 112 (12.4%) | 91 (10.6%) | 117 (13.0%) | 101 (10.9%) | 113 (10.8%) | 99 (10.8%) | 124 (11.1%) | 102 (9.9%) | 97 (9.1%) | 97 (9.6%) |  |
| Don't know | 269 (2.8%) | 20 (2.2%) | 21 (2.4%) | 33 (3.7%) | 30 (3.2%) | 32 (3.1%) | 25 (2.7%) | 39 (3.5%) | 29 (2.8%) | 36 (3.4%) | 4 (0.4%) |  |
| Don't wish to answer | 1 (0.0%) | 0 (0.0%) | 0 (0.0%) | 0 (0.0%) | 0 (0.0%) | 0 (0.0%) | 0 (0.0%) | 0 (0.0%) | 0 (0.0%) | 0 (0.0%) | 1 (0.1%) |  |
| Unknown | 402 | 42 | 53 | 45 | 45 | 35 | 39 | 38 | 47 | 56 | 2 |  |
| **Health insurance deductible** |  |  |  |  |  |  |  |  |  |  |  |  |
| none | 47 (0.5%) | 4 (0.4%) | 5 (0.5%) | 3 (0.3%) | 3 (0.3%) | 3 (0.3%) | 2 (0.2%) | 4 (0.3%) | 1 (0.1%) | 1 (0.1%) | 21 (2.1%) |  |
| 300 | 3,428 (33.8%) | 287 (30.5%) | 297 (32.6%) | 313 (33.1%) | 315 (32.5%) | 365 (34.0%) | 335 (35.2%) | 429 (37.1%) | 384 (35.7%) | 355 (31.8%) | 348 (34.5%) |  |
| 500 | 2,270 (22.4%) | 258 (27.4%) | 213 (23.4%) | 235 (24.8%) | 264 (27.2%) | 260 (24.2%) | 205 (21.5%) | 243 (21.0%) | 215 (20.0%) | 233 (20.9%) | 144 (14.3%) |  |
| 1000 | 434 (4.3%) | 45 (4.8%) | 38 (4.2%) | 47 (5.0%) | 58 (6.0%) | 49 (4.6%) | 32 (3.4%) | 53 (4.6%) | 39 (3.6%) | 48 (4.3%) | 25 (2.5%) |  |
| 1500 | 910 (9.0%) | 140 (14.9%) | 132 (14.5%) | 88 (9.3%) | 91 (9.4%) | 96 (8.9%) | 80 (8.4%) | 79 (6.8%) | 92 (8.6%) | 73 (6.5%) | 39 (3.9%) |  |
| 2000 | 236 (2.3%) | 19 (2.0%) | 14 (1.5%) | 27 (2.9%) | 23 (2.4%) | 31 (2.9%) | 22 (2.3%) | 28 (2.4%) | 28 (2.6%) | 27 (2.4%) | 17 (1.7%) |  |
| 2500 | 2,318 (22.8%) | 142 (15.1%) | 162 (17.8%) | 182 (19.2%) | 161 (16.6%) | 226 (21.0%) | 229 (24.1%) | 270 (23.3%) | 281 (26.1%) | 324 (29.0%) | 341 (33.8%) |  |
| Don't know | 487 (4.8%) | 46 (4.9%) | 51 (5.6%) | 52 (5.5%) | 54 (5.6%) | 45 (4.2%) | 47 (4.9%) | 51 (4.4%) | 35 (3.3%) | 55 (4.9%) | 51 (5.1%) |  |
| Don't wish to answer | 23 (0.2%) | 0 (0.0%) | 0 (0.0%) | 0 (0.0%) | 0 (0.0%) | 0 (0.0%) | 0 (0.0%) | 0 (0.0%) | 0 (0.0%) | 0 (0.0%) | 23 (2.3%) |  |
| Unknown | 16 | 1 | 1 | 0 | 4 | 2 | 1 | 0 | 0 | 1 | 6 |  |
| **BMI group** |  |  |  |  |  |  |  |  |  |  |  | 0.4 |
| Underweight | 939 (9.4%) | 64 (6.8%) | 71 (7.8%) | 84 (8.9%) | 97 (10.0%) | 99 (9.2%) | 93 (10.8%) | 117 (10.2%) | 113 (10.6%) | 107 (9.7%) | 94 (9.3%) |  |
| Healthy | 4,623 (46.1%) | 421 (45.0%) | 429 (47.4%) | 431 (45.8%) | 421 (43.5%) | 496 (46.2%) | 384 (44.7%) | 544 (47.3%) | 481 (45.0%) | 526 (47.5%) | 490 (48.4%) |  |
| Overweight | 3,153 (31.5%) | 322 (34.4%) | 286 (31.6%) | 307 (32.6%) | 311 (32.1%) | 340 (31.7%) | 278 (32.3%) | 350 (30.4%) | 324 (30.3%) | 340 (30.7%) | 295 (29.2%) |  |
| Obesity | 1,307 (13.0%) | 129 (13.8%) | 120 (13.2%) | 120 (12.7%) | 139 (14.4%) | 138 (12.9%) | 105 (12.2%) | 139 (12.1%) | 150 (14.0%) | 134 (12.1%) | 133 (13.1%) |  |
| Unknown | 147 | 6 | 7 | 5 | 5 | 4 | 93 | 7 | 7 | 10 | 3 |  |
| Smoker | 2,207 (21.9%) | 192 (20.4%) | 200 (22.0%) | 232 (24.5%) | 214 (22.0%) | 249 (23.1%) | 210 (22.1%) | 242 (21.0%) | 213 (19.8%) | 201 (18.0%) | 254 (27.1%) | <0.001 |
| Unknown | 88 | 1 | 2 | 1 | 2 | 0 | 1 | 2 | 0 | 2 | 77 |  |
| **Self;rated health** |  |  |  |  |  |  |  |  |  |  |  |  |
| Very good | 2,591 (26.7%) | 113 (23.1%) | 243 (26.7%) | 245 (25.9%) | 255 (26.3%) | 288 (26.8%) | 270 (28.3%) | 307 (26.5%) | 274 (25.5%) | 295 (26.4%) | 301 (29.8%) |  |
| Good | 5,355 (55.2%) | 290 (59.3%) | 491 (54.0%) | 503 (53.1%) | 534 (55.0%) | 581 (54.0%) | 515 (54.0%) | 644 (55.7%) | 597 (55.5%) | 616 (55.1%) | 584 (57.8%) |  |
| Average | 1,559 (16.1%) | 77 (15.7%) | 154 (16.9%) | 176 (18.6%) | 163 (16.8%) | 177 (16.5%) | 149 (15.6%) | 184 (15.9%) | 183 (17.0%) | 182 (16.3%) | 114 (11.3%) |  |
| Poor | 169 (1.7%) | 9 (1.8%) | 19 (2.1%) | 19 (2.0%) | 16 (1.6%) | 25 (2.3%) | 16 (1.7%) | 17 (1.5%) | 17 (1.6%) | 20 (1.8%) | 11 (1.1%) |  |
| Very poor | 30 (0.3%) | 0 (0.0%) | 2 (0.2%) | 4 (0.4%) | 3 (0.3%) | 4 (0.4%) | 3 (0.3%) | 5 (0.4%) | 4 (0.4%) | 4 (0.4%) | 1 (0.1%) |  |
| Unknown | 465 | 453 | 4 | 0 | 2 | 2 | 0 | 0 | 0 | 0 | 4 |  |

| **variable** | **Overall**, N = 10,169*^1^* | **2011**  N = 942*^1^* | **2012**  N = 913*^1^* | **2013**  N = 947*^1^* | **2014**  N = 973*^1^* | **2015**  N = 1,077*^1^* | **2016**  N = 953*^1^* | **2017**  N = 1,157*^1^* | **2018**  N = 1,075*^1^* | **2019**  N = 1,117*^1^* | **2024/25**  N = 1,015*^1^* | **p;value***^2^* |
| --- | --- | --- | --- | --- | --- | --- | --- | --- | --- | --- | --- | --- |
| **Dyslipidemia** |  |  |  |  |  |  |  |  |  |  |  | 0.052 |
| No | 7,393 (73.4%) | 667 (70.9%) | 644 (70.6%) | 690 (72.9%) | 701 (72.2%) | 808 (75.1%) | 703 (73.8%) | 866 (74.9%) | 773 (71.9%) | 835 (74.8%) | 706 (76.2%) |  |
| Yes | 2,679 (26.6%) | 274 (29.1%) | 268 (29.4%) | 257 (27.1%) | 270 (27.8%) | 268 (24.9%) | 249 (26.2%) | 290 (25.1%) | 302 (28.1%) | 281 (25.2%) | 220 (23.8%) |  |
| Unknown | 97 | 1 | 1 | 0 | 2 | 1 | 1 | 1 | 0 | 1 | 89 |  |
| **Diabetes** |  |  |  |  |  |  |  |  |  |  |  | 0.2 |
| No | 9,513 (94.4%) | 882 (93.6%) | 876 (96.2%) | 887 (93.7%) | 919 (94.5%) | 1027(95.4%) | 891 (93.6%) | 1091(94.4%) | 1005(93.5%) | 1058(95.0%) | 877 (94.7%) |  |
| Yes | 559 (5.6%) | 60 (6.4%) | 35 (3.8%) | 60 (6.3%) | 53 (5.5%) | 50 (4.6%) | 61 (6.4%) | 65 (5.6%) | 70 (6.5%) | 56 (5.0%) | 49 (5.3%) |  |
| Unknown | 97 | 0 | 2 | 0 | 1 | 0 | 1 | 1 | 0 | 3 | 89 |  |
| **Hypertension** |  |  |  |  |  |  |  |  |  |  |  | 0.005 |
| No | 8,126 (80.6%) | 728 (77.4%) | 719 (78.9%) | 757 (80.1%) | 791 (81.5%) | 844 (78.4%) | 699 (80.0%) | 956 (82.6%) | 864 (80.6%) | 929 (83.3%) | 839 (82.7%) |  |
| Yes | 1,951 (19.4%) | 213 (22.6%) | 192 (21.1%) | 188 (19.9%) | 180 (18.5%) | 233 (21.6%) | 175 (20.0%) | 201 (17.4%) | 208 (19.4%) | 186 (16.7%) | 175 (17.3%) |  |
| Unknown | 92 | 1 | 2 | 2 | 2 | 0 | 79 | 0 | 3 | 2 | 1 |  |
| *^1^* Mean (SD); n (%) | | | | | | | | | | | | |
| *^2^* Kruskal;Wallis rank sum test; Pearson’s Chi;squared test | | | | | | | | | | | | |

**Supplementary Table 2. Average prevalence of forgoing healthcare for financial reasons, with average changes of premiums (Bus Sante study, Geneva, Switzerland, 2011 to 2025)**

| **Year** | **Overall** | **Female** | **Male** | **25-34** | **35-49** | **50-64** | **65 and above** | **Premium in CHF** | **Percent increase premium** |
| --- | --- | --- | --- | --- | --- | --- | --- | --- | --- |
|  | % | % | % | % | % | % | % | CHF | Δ% |
| 2011 | 15.8 | 16.9 | 14.6 | 11.1 | 17.8 | 15.7 | 9.4 | 384 | 0.0 |
| 2012 | 17.2 | 19.6 | 14.9 | 21.5 | 19.7 | 14.4 | 12.1 | 408 | 6.2 |
| 2013 | 18.9 | 17.6 | 20.4 | 26.8 | 21.0 | 13.6 | 12.8 | 420 | 9.3 |
| 2014 | 15.4 | 17.5 | 12.9 | 20.1 | 17.6 | 12.8 | 7.7 | 437 | 13.8 |
| 2015 | 16.8 | 18.9 | 14.6 | 19.6 | 18.7 | 18.1 | 5.3 | 459 | 19.5 |
| 2016 | 17.0 | 18.3 | 15.6 | 21.2 | 18.6 | 14.4 | 12.3 | 482 | 25.5 |
| 2017 | 16.0 | 17.3 | 14.6 | 20.2 | 16.8 | 14.5 | 11.3 | 503 | 31.0 |
| 2018 | 18.6 | 20.1 | 17.1 | 24.8 | 18.8 | 18.3 | 12.7 | 527 | 37.2 |
| 2019 | 19.8 | 21.2 | 18.2 | 27.8 | 21.2 | 18.7 | 9.4 | 546 | 42.2 |
| 2024-25 | 25.8 | 28.6 | 23.0 | 38.4 | 28.8 | 16.1 | 13.4 | 622 | 61.9 |

* Δ% Percent increase

Data were missing between 2020-2023 due to the COVID-19 pandemic

**Supplementary Table 3. Associations between socioeconomic or health determinants and forgoing healthcare for financial reasons stratified by deductible (Bus Sante study, Geneva, Switzerland, 2011 to 2025)**

| **variable** | **Low deductible**  **N=5,642** |  | **Middle deductible**  **N=1,338** |  | **High deductible**  **N=2,513** |  |
| --- | --- | --- | --- | --- | --- | --- |
|  | aOR [95%CI] | p-value | aOR [95%CI] | p-value | aOR [95%CI] | p-value |
| **Survey year** | 1.05 [1.03;1.08] | <0.001 | 1.04 [0.98;1.1] | 0.188 | 1.10 [1.07;1.14] | <0.001 |
| **Age groups** |  |  |  |  |  |  |
| 65 years and older | Ref |  | Ref |  | Ref |  |
| 25-34 | 2.42 [1.70;3.44] | <0.001 | 4.02 [1.79;9.04] | 0.001 | 2.70 [1.34;5.42] | 0.005 |
| 35-49 | 2.29 [1.70;3.09] | <0.001 | 2.22 [1.09;4.53] | 0.028 | 1.92 [0.97;3.77] | 0.060 |
| 50-64 | 1.56 [1.19;2.06] | 0.001 | 2.02 [1.02;3.99] | 0.043 | 1.37 [0.69;2.73] | 0.375 |
| **Sex** |  |  |  |  |  |  |
| Male | Ref |  | Ref |  | Ref |  |
| Female | 1.14 [0.95;1.37] | 0.154 | 1.23 [0.85;1.79] | 0.269 | 1.38 [1.09;1.74] | 0.007 |
| **Income** |  |  |  |  |  |  |
| 7000-9499 | Ref |  | Ref |  | Ref |  |
| <3000 | 2.52 [1.76;3.60] | <0.001 | 1.60 [0.66;3.88] | 0.300 | 1.96 [1.10;3.47] | 0.022 |
| 3000-4999 | 1.98 [1.51;2.61] | <0.001 | 2.24 [1.17;4.29] | 0.014 | 1.51 [1.00;2.29] | 0.05 |
| 5000-6999 | 1.27 [0.98;1.65] | 0.067 | 1.97 [1.14;3.41] | 0.016 | 1.17 [0.82;1.67] | 0.39 |
| 9500-13000 | 0.63 [0.46;0.85] | 0.002 | 0.97 [0.54;1.74] | 0.918 | 0.79 [0.56;1.12] | 0.192 |
| >13000 | 0.31 [0.21;0.46] | <0.001 | 0.59 [0.31;1.13] | 0.112 | 0.44 [0.31;0.65] | <0.001 |
| **Nationality** |  |  |  |  |  |  |
| Swiss | Ref |  | Ref |  | Ref |  |
| Non swiss | 1.00 [0.83;1.20] | 0.964 | 0.98 [0.66;1.44] | 0.900 | 1.10 [0.87;1.39] | 0.448 |
| **Education** |  |  |  |  |  |  |
| Primary | Ref |  | Ref |  | Ref |  |
| Secondary | 1.31 [1.01;1.69] | 0.045 | 1.57 [0.79;3.13] | 0.200 | 2.21 [1.23;3.97] | 0.008 |
| Tertiary | 1.58 [1.20;2.08] | 0.001 | 1.90 [0.94;3.83] | 0.074 | 2.46 [1.39;4.37] | 0.002 |
| **Civil status** |  |  |  |  |  |  |
| Married/in couple | Ref |  | Ref |  | Ref |  |
| Single | 0.64 [0.49;0.83] | 0.001 | 0.78 [0.46;1.31] | 0.343 | 0.60 [0.44;0.83] | 0.002 |
| Divorced living alone | 1.43 [1.14;1.80] | 0.005 | 1.16 [0.69;1.96] | 0.567 | 1.10 [0.73;1.66] | 0.643 |
| Divorced/in couple | 1.17 [0.76;1.80] | 0.488 | 0.46 [0.17;1.22] | 0.120 | 1.41 [0.74;2.69] | 0.297 |
| Widowed living alone | 0.72 [0.42;1.24] | 0.234 | 2.15 [0.81;5.74] | 0.126 | 0.86 [0.32;2.32] | 0.762 |
| Widowed/in couple | 0.61 [0.13;2.80] | 0.526 | NA |  | NA |  |

| **Subsidies** |  |  |  |  |  |  |
| --- | --- | --- | --- | --- | --- | --- |
| No | Ref |  | Ref |  | Ref |  |
| Yes | 1.36 [1.09;1.69] | 0.006 | 1.58 [0.94;2.68] | 0.086 | 1.52 [1.11;2.08] | 0.008 |
| **Complementary insurance** |  |  |  |  |  |  |
| Yes | Ref |  | Ref |  | Ref |  |
| No | 1.55 [1.29;1.86] | <0.001 | 1.64 [1.12;2.38] | 0.010 | 1.65 [1.29;2.09] | <0.001 |
| **Smoking** |  |  |  |  |  |  |
| No | Ref |  | Ref |  | Ref |  |
| Yes | 1.39 [1.15;1.68] | 0.001 | 1.35 [0.91;2.01] | 0.139 | 1.58 [1.22;2.05] | 0.001 |
| **Self-rated health** |  |  |  |  |  |  |
| Very good | Ref |  | Ref |  | Ref |  |
| Good | 1.41 [1.11;1.78] | 0.004 | 2.04 [1.25;3.35] | 0.005 | 1.59 [1.24;2.04] | <0.001 |
| Average | 2.46 [1.88;3.22] | <0.001 | 3.10 [1.71;5.64] | <0.001 | 3.82 [2.63;5.54] | <0.001 |
| Poor | 3.79 [2.28;6.30] | <0.001 | 11.75 [3.23;42.69] | <0.001 | 12.37 [3.57;42.91] | <0.001 |
| Very poor | 7.30 [2.22;23.9] | 0.001 | NA |  | NA |  |
| **BMI** |  |  |  |  |  |  |
| Healthy | Ref |  | Ref |  | Ref |  |
| Underweight | 0.95 [0.70;1.28] | 0.717 | 0.91 [0.48;1.73] | 0.769 | 1.38 [0.98;1.92] | 0.062 |
| Overweight | 1.01 [0.83;1.24] | 0.905 | 1.28 [0.85;1.92] | 0.236 | 0.99 [0.76;1.30] | 0.955 |
| Obesity | 1.13 [0.87;1.45] | 0.362 | 1.60 [0.91;2.82] | 0.103 | 0.99 [0.64;1.54] | 0.970 |
| **Dyslipidemia** |  |  |  |  |  |  |
| No | Ref |  |  |  |  |  |
| Yes | 0.87 [0.72;1.06] | 0.179 | 0.66 [0.41;1.07] | 0.089 | 0.83 [0.61;1.13] | 0.243 |
| **Diabetes** |  |  |  |  |  |  |
| No | Ref |  |  |  |  |  |
| Yes | 1.31 [0.95;1.79] | 0.098 | 1.83 [0.75;4.47] | 0.188 | 0.89 [0.41;1.93] | 0.761 |
| **Hypertension** |  |  |  |  |  |  |
| No | Ref |  |  |  |  |  |
| Yes | 0.83 [0.66;1.05] | 0.113 | 1.16 [0.68;1.98] | 0.585 | 0.64 [0.41;1.00] | 0.051 |

Adjusted for age, sex, education, income and survey year

Deductible was considered as low (300-500CHF), middle (1000-1500CHF) and high (2000-2500CHF)

**Supplementary Table 4. Model performance comparison for predictions of changes in insurance premium and forgoing healthcare (Bus Sante study, Geneva, Switzerland, 2011 to 2025)**

| **Variable** | **Model** | **AIC** | **BIC** | **RMSE** | **MAE** | **R²** | **Adj R²** |
| --- | --- | --- | --- | --- | --- | --- | --- |
| Insurance Premium | LOESS | 42.94 | 47.74 | 2.298 | 1.891 | 0.9961 | - |
| Insurance Premium | Natural Spline | 49.40 | 54.38 | 2.678 | 2.219 | 0.9947 | 0.9933 |
| Insurance Premium | Polynomial (degree 3) | 58.16 | 62.14 | 3.504 | 3.068 | 0.9909 | 0.9892 |
| Insurance Premium | Polynomial (degree 2) | 80.97 | 83.96 | 6.517 | 5.479 | 0.9686 | 0.9649 |
| Insurance Premium | Holt Exponential Smoothing | 95.27 | 98.26 | 9.316 | 5.396 | 0.9357 | - |
| Insurance Premium | Linear | 99.59 | 101.58 | 10.911 | 8.345 | 0.9118 | 0.9069 |
| Forgoing Healthcare | LOESS | 67.34 | 70.91 | 6.148 | 5.010 | 0.9878 | - |
| Forgoing Healthcare | Natural Spline | 67.59 | 71.45 | 6.047 | 4.926 | 0.9882 | 0.9840 |
| Forgoing Healthcare | Polynomial (degree 3) | 66.76 | 69.85 | 6.273 | 5.173 | 0.9873 | 0.9842 |
| Forgoing Healthcare | Polynomial (degree 2) | 68.01 | 70.32 | 6.943 | 5.663 | 0.9845 | 0.9821 |
| Forgoing Healthcare | Holt Exponential Smoothing | 86.22 | 88.54 | 12.268 | 8.125 | 0.9516 | - |
| Forgoing Healthcare | Linear | 100.91 | 102.46 | 20.667 | 18.214 | 0.8626 | 0.8528 |

AIC: Akaike Information Criterion; BIC: Bayesian Information Criterion; RMSE: Root Mean Square Error; MAE: Mean Absolute Error; R²: Coefficient of Determination; Adj R²: Adjusted Coefficient of Determination; LOESS: Locally Estimated Scatterplot Smoothing.

**Supplementary Figure 1. Trends of forgoing healthcare for financial reasons stratified by income categories (Bus Sante study, Geneva, Switzerland, 2011 to 2025)**

Results show the percentage of forgoing healthcare per income group every year between 2011 and 2025. Results for 2020-2023 were missing due to the COVID-19 pandemic.

**Supplementary Figure 2. LOESS Model validation analysis (Bus Sante study, Geneva, Switzerland, 2011 to 2025)**

LOESS: Locally Estimated Scatterplot Smoothing
