# Supplementary figures and images for: Forgoing Healthcare and Insurance Premiums Trends: A 15-Year Population-Based Study in Geneva, Switzerland
Source: Int J Public Health. 2025 Nov 26;70:1609027. doi: 10.3389/ijph.2025.1609027 (PMC12689442; doi:10.3389/ijph.2025.1609027)

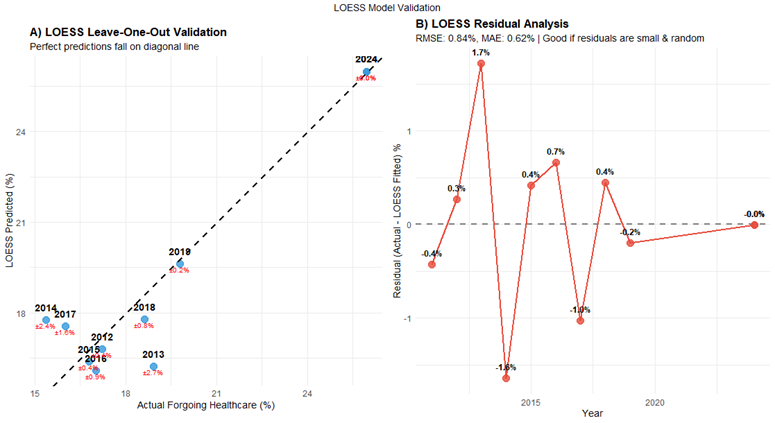

Supplement: Supplementary file 2 [file Image2.png]

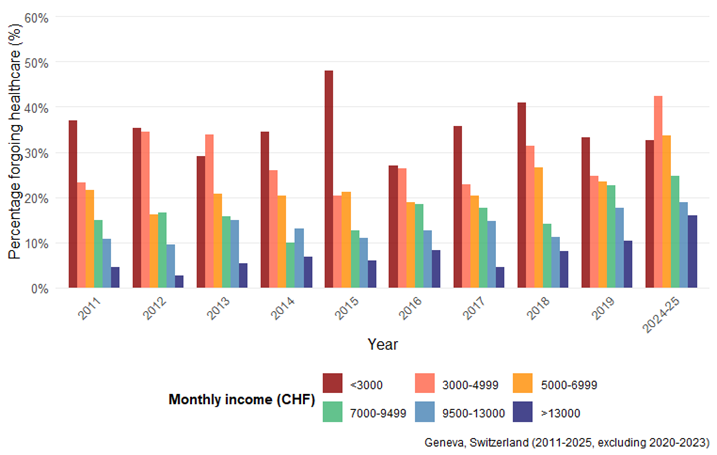

Supplement: Supplementary file 3 [file Image1.png]
